# The COVID-19 Crisis: An Opportunity to Integrate Food Democracy into Post-Pandemic Food Systems

**DOI:** 10.1017/err.2020.40

**Published:** 2020-04-22

**Authors:** Ludivine PETETIN

**Affiliations:** * Senior Lecturer in Law, Cardiff University, UK; email: PetetinL@cardiff.ac.uk.

## Abstract

The world economy is sliding yet into another recession (having arguably barely recovered from the previous economic downturn) due to the worldwide pressures and tensions created by the COVID-19 pandemic.^1^ With most countries in the world under lockdown (or in similar situations), almost all food is now consumed in the household. Arguably, agricultural producers and the retail industry appear to be the best placed to weather the storm in order to respond to such a change in demand. However, this is overly simplistic. Recent news of empty shelves in supermarkets whilst dairy farmers have been forced to pour milk down the drain have gone viral.

## I. Introduction

The world economy is sliding yet into another recession (having arguably barely recovered from the previous economic downturn) due to the worldwide pressures and tensions created by the COVID-19 pandemic.^1^ With most countries in the world under lockdown (or in similar situations), almost all food is now consumed in the household. Arguably, agricultural producers and the retail industry appear to be the best placed to weather the storm in order to respond to such a change in demand. However, this is overly simplistic. Recent news of empty shelves in supermarkets whilst dairy farmers have been forced to pour milk down the drain have gone viral.

Through the ongoing disruptions within the food supply chain at both international and national levels, the pandemic offers a singular circumstance to rethink food provision and build resilient, sustainable and democratic agri-food systems. In particular, food democracy (sometimes also called “food citizenship”^2^) provides a model for multilevel food governance to be followed post-coronavirus where a wide range of actors are involved in the design and delivery of future food systems. Consequently, this article highlights the challenges faced by the international trade in agricultural products (Section II) before indicating the dysfunctions within food supply chains from a national viewpoint (Section III) and how these issues (especially around food disruptions) can cause consumers to consider the sources of their food more carefully.^3^ Most importantly, the pandemic emphasises the urgency to rebuild food systems based on a food democracy model (Section IV) before considering future developments (Section V).

## II. Risks to global food trade and supply chains

At the time of writing, the spread of the virus is sweeping across the world, one country after another, with increasing intensity. The World Trade Organization (WTO) has warned that global trade could diminish by between 13% and 32% in 2020 due to the COVID-19 outbreak, with North America and Asia being hit the hardest, but it foresees a recovery in 2021 – albeit dependent on the length of the pandemic.^4^ The supply of food is becoming increasingly difficult – highlighting the fragility of global food supply chains – thereby raising concerns of food insecurity.

Lockdowns worldwide have consequences on harvesting, production, processing, transport and logistics more generally for multiple reasons. The volume of production is negatively impacted globally. The number of employees who are self-isolating or unwell is increasing. Workplaces and spaces are being redesigned to accommodate social distancing requirements and putting in place more stringent handling procedures (including the purchase of employee personal protection equipment), whilst others are either closing for extended periods of time due to longer and deeper cleans or are planning temporary shutdowns due to staff shortages, in particular meat processing plants.^5^ There is also an issue of increased food waste when containers or trucks are held up at borders. These factors are resulting in the rise of raw material prices due to discrepancies in supply and demand. This is the case in India where the prices of rice, wheat flour and pulses have risen.^6^

Another issue that impacts on the production of food globally is the lack of seasonal workers to pick the fruits and vegetables in the fields due to border restrictions and travel bans. These agricultural workers are paramount to ensuring national and international food security, as they undertake highly skilled, difficult work. The European Union (EU), the UK, Canada and the USA are some of the countries particularly affected.^7^ In the UK, around 80,000 seasonal employees are annually employed in the horticultural industry.^8^ Usually, this workforce originates from other EU countries, especially Eastern European countries.^9^ To respond to these concerns, Romania recently lifted its border restrictions temporarily to allow agricultural workers to work abroad. Many are now working in the UK or Germany – the latter needs 300,000 workers for the harvest season, such as to pick asparagus.^10^

Some States are also considering export restrictions, whilst others have already put them in place to ensure stability and food security within their territory. These measures could create global food shortages, especially in poorer countries.^11^ Some key staple foods are more affected than others.^12^ For example, one of the top countries to produce rice, Vietnam, has temporarily ceased sales of new shipments.^13^ Grain exporters, including Russia and Kazakhstan, have also suspended exports to ensure that their populations are nourished.^14^ States stockpiling agricultural products could trigger increased price volatility. Such events recall the 2007–2008 world food prices peak and the resulting food insecurity crisis.^15^

These restrictions are multiplying across the world despite a Joint Statement of the WTO, the UN Food and Agriculture Organization (FAO) and the World Health Organization (WHO) advising against such measures and stressing the importance to “show solidarity, act responsibly and adhere to our common goal of enhancing food security, food safety and nutrition and improving the general welfare of people around the world”.^16^ If the uptake of these export restrictions keeps growing, they could have ripple effects on the global food supply chain. They could contribute to food insecurity worldwide – potentially increasing insecurity in the longer term in the countries having imposed these protective measures. Despite the disruptions highlighted so far, the supply to current global markets remains adequate.^17^ Nonetheless, a combination of all of these changes leads to increased prices for food businesses. These increased costs will be passed on to the costumers in the future (if not having done so already).^18^

With the current context, the danger of going back to the old habits of high-input, high-output modes of production – whereby external inputs (herbicides, pesticides, fertilisers) are utilised to increase the levels of food production – is growing and threatening the continuing turn towards sustainable agriculture.^19^ However, at most this would likely be a short-term fix for food security, as sustainability is required in the long term.^20^ The drive for sustainable agriculture must not be rolled back and should be prioritised, with renewed emphasis on tackling climate change and moving agriculture towards net-zero emissions by 2050.^21^ Furthermore, many synthetic inputs are produced in China,^22^ and due to the Chinese lockdown, their production has been disrupted, which could lead to some products being temporarily discontinued and intensification being challenged in the short term. The temptation to revert back to less environmentally friendly practices or to embrace novel food technologies, such as the genetic modification of crops and animals or animal cloning, should be carefully monitored and assessed. More positively, the COVID-19 crisis provides an opportunity to reconsider current agricultural approaches and to farm using greener techniques. The pandemic puts the resilience of the existing dominant food systems under strain at both international and national levels.

## III. Disrupted food systems: internal perspectives

Many governments remain uncertain about how to handle the virus and contain it as it spreads. Due to this and fears over the potential impacts of the virus on public health and on food supply chains, citizens rushed to the shops to stockpile food across the globe – before lockdowns were fully in place and despite governments repeatedly asserting that there would be no food shortages.^23^ This led poorer and vulnerable people to have limited access to staple foods.

In the medium to long term, as unemployment rates grow worldwide and household income decreases, citizens will prioritise their food spending. The percentage of household income dedicated to food will keep increasing, whilst the price of food will keep rising due to the disruption in food supply and the increased demand by citizens.^24^ Relying on supplies from food banks will become a necessity for many, whilst contributions are problematically diminishing.^25^ Programmes and schemes ought to be put in place to support struggling households. An international food crisis is also a possibility.^26^ Some organisations are calling for a “global stimulus package to fight a COVID-19 hunger crisis”,^27^ whilst the G20 suspended debt payments for low-income nations.^28^ Citizens in low- and middle-income countries should not be forgotten. The ongoing developments are highlighting the strong links between food security and public health and could deepen global inequalities.

The main source of food and agricultural products for citizens is now retailers, mostly supermarkets. Queuing to enter shops and supermarkets has become the new norm, as well as rationing. Supermarkets have placed limits on how many items of the same product people can buy, including milk, toilet paper and flour. They also have restricted the number of customers on the shop floor at any one time and implemented a “one in, one out” system. Because citizens are either on lockdown restrictions or have been told to stay at home as much as possible, more online orders are being placed, with citizens choosing home deliveries or “click and collect”^29^ options. Again, supermarkets have had to adapt to the increasing online demand. Today, shop floors are packed with employees picking the products ordered by consumers to get online orders ready. Many supermarket chains have recruited staff to help in this effort to “feed the nation”.^30^ Food retailers are booming in terms of employee numbers, public orders and consumption.

From a citizen perspective, there seem to be “shortages”. Some shelves are rarely replenished, and there is a lack of availability of certain products, especially baby formula and flour. In contrast, it has been reported throughout the world that dairy farmers are pouring perfectly safe milk down the drain.^31^ Similarly, flour mills have been running at capacity for the last few weeks, yet flour does not seem to be back on the shelves.^32^ This contrasts with the earlier statement that agricultural producers would be benefitting from the situation.

So, what is the problem? It is not due to the lack of demand in shops or the availability of the agricultural commodity, but to the (dis)organisation of the supply chains. To put it simply, the food supply chain is split between providing food for retail and for food service businesses (restaurants, cafes, fast food chains, delis, bars and pubs). The latter often buy in bulk (often tankers, large containers or bags), whilst the former only require reduced quantities in smaller packaging. The ongoing situation in shops indicates that the food supply chain is not equipped to adapt quickly to modified demand both in terms of increased quantities and sourcing the relevant containers or packaging. The availability of smaller containers for flour bags, milk cartons and even tinned food is insufficient to cope with the expanded demand in retail.^33^ There is a lack of capacity for this type of demand. Another difference between retail and food service markets is the nature of the foods sold: the latter often attract more high-end, gourmet products, such as prime cuts of meat, than the former, thus it is difficult to shift products from one to the other. These major shifts in consumption patterns are impacting on the resilience of all of the industries involved in food supply chains.

The loss of demand from all food service businesses due to the often imposed shutdowns is modifying the location and place of food consumption. Sadly, this shortfall is often not compensated by a corresponding increase in retail consumption.^34^ In the USA, the International Dairy Foods Association (IDFA) estimates that “10% of the nation’s milk supply is without a home for the foreseeable future”.^35^ Finding a new home for lost demand is proving difficult. The markets are having difficulties in adjusting to this new normal. Such a reorganisation of food production takes up many resources in terms of time and costs because the industry works on well-established networks and food supply chains.

The COVID-19 crisis indicates that the “just-in-time characteristic of the food supply chain”^36^ and its logistics cannot easily cope with disruptions and turbulence. Just-in-time supply chains aim to guarantee the freshness of products and to reduce waste, but they require the coordination and effectiveness of each actor along the chain. However, when one of its elements is negatively affected, it has consequences on others.^37^ When multiple actors are impacted, the long-lasting ramifications can be felt from the primary producer to the citizen.

The lack of capacity for adaptation and change in the food supply chain highlights the relatively weak role of the farmer in food systems, despite the farmer being the one who actually provides the raw agricultural product. The farmer is only one (small) actor and depends on the other actors in the food supply chain to sell their produce. The ongoing issues reinforce the calls to address concerns about the unfairness within agri-food supply chains.^38^ A stronger role for farmers in food systems is crucial to an approach based on food democracy.

## IV. Food democracy as a model for multilevel food governance post-pandemic

The pandemic indicates the urgency of rethinking the food system and its characteristics. There appears to be limited food security, as is demonstrated when the system is put under pressure. There is little resilience or flexibility, and this is partially linked to the weak position of farmers and the existence of long food supply chains. Often, citizens do not feel in control of the international food supply chain or food provenance. They feel like food choice is imposed by the supply chain. For example, in Wales it can be difficult from time to time to buy locally sourced Welsh produce, especially lamb, since 40% of it is exported – mostly to the EU (even when in season).^39^

A food democracy model gives citizens opportunities to actively participate in how sustainable food systems are constructed in order to enable alternative perspectives on how food should be produced and consumed. The term “food democracy” was coined in the 1990s by Lang.^40^ Food democracy creates a framework that is about building a transformative, alternative food system to empower citizens through choices and to allow them to find greater satisfaction in a food system that reflects shared values underpinning societies and that sells sustainable produce that people want.^41^ These individual choices about where people buy food demonstrate “the degree of control consumers can exert” on food systems.^42^ This model for multilevel food governance can be characterised as citizens wanting “better food, more information and choices, and preference for local action and personal involvement”,^43^ and it ought to be followed when rebuilding food supply chains.

This section draws upon the work of Petetin on food democracy. She identifies four essential characteristics of a food democracy model (that builds upon traditional concepts of democracy); attributes that can indeed be found in the ongoing developments triggered by COVID-19:

i. *True information, genuine choice and alternative products being offered to consumers;*
ii. *Upstream engagement and bottom up approach in the decision-making process;*
iii. *Good health, food safety, sustainable agriculture and environmental protection, improvement of the rights of farmers and agricultural workers and their opportunities; and,*
iv. *Restoration of faith and trust in the food system, its institutions and in farmers.* ^44^

Since the lockdowns have been put in place, some small, family farms have been thriving. Vegetable and fruit box schemes that sell directly to citizens have boomed, especially organic ones.^45^ Whilst costs are similar to normal shops, they enable access without queuing (when available for order). It is also possible that citizens perceive this as a “treat” since they can no longer go to restaurants. Many schemes were not able to accept new customers temporarily due to the swift increase in demand for local produce. The situation was similar for some local butchers who had to temporarily close^46^ in order to move to deliveries and collections, whilst giving them time to improve their websites in order to enable such changes. It appears that some small local shops and agricultural producers are showing a certain degree of adaptation and resilience to cope with feeding the local population (characteristic ii).

By buying fruit and vegetable boxes and going to their local butchers and bakers, citizens are taking charge of their food consumption, choosing what they want to eat and building a democratic agri-food system (characteristics i and iv). This is different from going to the restaurant or getting takeaways since citizens often do not know the provenance of the foods from these services. These alternative ways of shopping have turned “consumers” into “active citizens” who carefully choose what is on their plate.^47^ Furthermore, in a recent blogpost, Hawkes sets the scene for a new normal post-pandemic based on eating well and embracing the opportunity “to open the floodgates to nutritious foods – taking unhealthy foods and their relentless promotion out of the spotlight”.^48^ Buying locally and directly from farmers arguably means the consumption of healthier, more nutritious food and could have a positive impact on public health. Shorter supply chains can mean less packaging and processing and reduce food miles (characteristic iii) and will strengthen the importance of farmers in food systems (characteristic iv). It is to be hoped that this renewed interest for locally grown and reared food will last post-pandemic and will durably encourage more sustainable and healthier lifestyles.

More citizens have also taken up gardening and have been buying seeds and thus improving their self-sufficiency and increasing national food security.^49^ This decision by citizens to be in charge of their food choices reinforces the multi-actor and multilevel framework underpinning food democracy.

In early April 2020, it was reported that 26,000 British citizens had positively responded to the British shortfall in agricultural workers.^50^ This was hailed as a positive, welcome move (characteristic iv). It has now emerged that only 6000 British citizens were interviewed to undertake these jobs.^51^ The main reasons given for not progressing with the applications were the length of the contract, the location of the farm and the inability to work full-time due to caring duties. A UK cross-industry online platform has now been created to link fruit and vegetables farms to job-seekers.^52^ In order to reduce the shortfall, Romanian agricultural workers were flown into the UK.^53^ More citizens need to come forward to ensure that fruits and vegetables are not left to rot in the fields, especially to pick labour-intensive fruits such as strawberries. Otherwise, this could lead to the reduced availability of fresh produce on the shelves.

Here, the crisis provides the chance to train and develop skills for local workers. They ought to be perceived as “essential workers” who should be valued, allowed to work in good conditions and receive decent wages in line with food democracy characteristics.^54^ This could ensure a reliable and long-lasting workforce in the future for farmers and of employment for the local population (characteristic iii).

The COVID-19 crisis highlights the centralised^55^ characteristics of food systems – some of which heighten vulnerabilities and lead to insecurity. Food democracy provides a decentralised framework for food systems that offers the opportunity to redesign the food supply chain to focus to a greater extent on sustainability and local and regional production by building stronger direct links between retailers and local farms. Democratised agri-food systems put to the fore different sustainable types of farming practices and construct alternative models of production, distribution and retailing that offer choices and alternatives for people with various incomes.

Overall, a shift towards fulfilling the criteria of a food democracy model can be observed. It is, however, not guaranteed that these positive shifts will be maintained post-pandemic. Further steps are required to strengthen democratic agri-food systems. In particular, financial agricultural support needs to be redesigned to support small, family farms to a greater extent in their role as local food producers. Farms, as small and medium-sized enterprises, are essential to rural vitality and the local economy. They can facilitate the establishment and flourishing of businesses and economic activities such as rural tourism, cultural services, recreational activities and access to local products, thereby providing local employment. Supporting the diversification of holdings and the development of cooperatives to take up not only production, but also processing and packaging on a small scale could improve the finances of their businesses, enhance access to food and strengthen a democratic agri-food system. Furthermore, small, family farms are stewards of the countryside and tend to be environmentally friendly by creating greater biodiversity and a wider range of habitats on the land through the use of extensive, alternative agricultural farming practices,^56^ such as agroecology and agroforestry,^57^ contributing to the strengthening of agri-food systems (characteristic iii).^58^

Another step could be to integrate more fully food and agricultural policies. The current Common Agricultural Policy (CAP) reform and the ongoing UK and devolved consultations on future agricultural policies post-EU exit (including the 2020 Agriculture Bill) could create such frameworks and mechanisms. Currently, agricultural policy and food strategies are formulated in isolation from each other.^59^ The European Green Deal, which includes a “Farm to Fork Strategy” that aims to strengthen the production of safe, nutritious food of high quality as well as increasing its sustainability,^60^ will partially respond to this siloed approach. However, a more holistic approach between agriculture and food underpinned by a strong community focus should be adopted. The pandemic highlights the importance of formulating policies (and resulting bills) that combine both agricultural and food aspects.

## V. Further food for thought

With the breakdown of international trade because States are closing their borders to keep food supplies within their territory and companies either are closing or are not fully operational, food provisioning is becoming increasingly difficult. Existing weaknesses and tensions in complex, interconnected and global food supply chains have been exacerbated by COVID-19. Despite the multiplicity of farmers, bottlenecks in the food supply chain are often due to consolidation and integration within the industry because of the presence of a limited number of processing plants, abattoirs and retailers. In the UK, Brexit will potentially add another layer to the ongoing crisis since the future trading relationship with the EU remains uncertain.

With the current changes in the food supply chain, food citizens are providing the impetus for change and have become empowered to influence and nurture better food provisioning to “create a more sustainable and just society where the public can actively participate in the decision-making process for foods”.^61^ The pandemic is creating a rare opportunity for radical change^62^ to transform food supply chains and build resilient, sustainable and democratic agri-food systems (including in the design of financial agricultural support). Post-pandemic, food democracy generates a strong framework for multilevel food governance by putting the emphasis on local and regional production that encourages the consumption of seasonal and healthy produce (in combination with longer food supply chains and the provisioning of sustainable products), employing local agricultural workers and establishing better relationships between producers and retailers, whilst ensuring that the security and diversity of food are maintained. These modifications triggered by local, national and international factors ought to be reflected in the future policies of individual States and international organisations integrating food and agriculture, including the ongoing CAP reform, the 2020 UK Agriculture Bill and the Welsh, Northern Irish and Scottish visions for future agri-food systems.

## Footnotes

1 The International Monetary Fund forecasts the worst recession since the Great Depression. See, eg, G Gopinath, “The Great Lockdown: Worst Economic Downturn since the Great Depression”, *IMFBlog*, 14 April 2020 <https://blogs.imf.org/2020/04/14/the-great-lockdown-worst-economic-downturn-since-the-great-depression>.

2 See, eg, Food Ethics Council, “Harnessing the Power of Food Citizenship” (2019) <https://www.foodethicscouncil.org/app/uploads/2019/10/Harnessing-the-power-of-food-citizenship_Food_Ethics_Council_Oct-2019.pdf>.

3 Where relevant, examples originating from the UK will be utilised.

4 Director General Azevêdo, Trade Forecast Press Conference (WTO, 8 April 2020) <https://www.wto.org/english/news_e/spra_e/spra303_e.htm>.

5 ZB Wolf, “It’s Time to Talk More Seriously About the Food Supply”, *CNN*, 14 April 2020 <https://edition.cnn.com/2020/04/14/politics/what-matters-april-13/index.html>.

6 A Laskar, “Prices of Rice, Pulses Spike in Mumbai”, *Livemint*, 12 April 2020 <https://www.livemint.com/news/india/covid-19-crisis-mumbai-daal-chawal-prices-rise-11586512016776.html>.

7 See, eg, N Foote, “COVID-19 Measures Could Cause ‘Devastating’ Labour Shortage in EU Farming”, *EURACTIV.com*, 25 March 2020 <https://www.euractiv.com/section/agriculture-food/news/covid-19-measures-could-cause-devastating-labour-shortage-in-eu-farming>; S Thevenot, “Agriculture Labour Shortage Feared Amid COVID-19 Travel Bans”, *Canadian Immigration Newsletter*, 17 March 2020 <https://www.cicnews.com/2020/03/agriculture-labour-shortage-feared-amid-covid-19-travel-bans-0313913.html#gs.3dvf1b>.

8 Country Land and Business Association, “Government Clarifies Furloughing Rules after CLA Lobbying”, 7 April 2020 <https://www.cla.org.uk/government-clarifies-furloughing-rules-after-cla-lobbying>. Due to Brexit and the end of the EU principle of free movement of persons, the situation in the UK to recruit agricultural workers was already going to be difficult. See, eg, L Petetin, “Cloud Nine or Down to Earth? The Implications of a No Deal Brexit on Agriculture”, *The UK in a Changing Europe*, 15 August 2019 <https://ukandeu.ac.uk/cloud-nine-or-down-to-earth-the-implications-of-a-no-deal-brexit-on-agriculture>.

9 S Lakner, “Effects of Coronavirus on Agricultural Production – A First Approximation (part 2)”, *Arc 2020*, 6 April 2020 <https://www.arc2020.eu/effects-of-coronavirus-on-agricultural-production-a-first-approximation-part-2>.

10 A Kühnel, “Germany Drafts Romanian Farm Labor for Coronavirus Pandemic”, *Deutsche Welle*, 8 April 2020 <https://www.dw.com/en/germany-drafts-romanian-farm-labor-for-coronavirus-pandemic/a-53066735>.

11 Under WTO law, such export restrictions could potentially be justified under Art XX (General Exceptions) (in particular Art XX (b) on measures to protect human health) and Art XXI (Security Exception) of the 1994 General Agreement on Tariffs and Trade (GATT). The issue of their legality is beyond the scope of this manuscript. For more, see, eg, V Lapa, “GATT Article XXI as a Way to Justify Food Trade Restrictions Adopted as a Response to COVID-19?”, *Regulating for Globalization*, 10 April 2020 <http://regulatingforglobalization.com/2020/04/10/gatt-article-xxi-as-a-way-to-justify-food-trade-restrictions-adopted-as-a-response-to-covid-19>.

12 The International Food Policy Research Institute has developed an excellent tracker of food export restrictions during the crisis; see D Laborde, “Food Export Restrictions during the Covid-19 Crisis”, *International Food Policy Research Institute*, 2020 <https://public.tableau.com/profile/laborde6680#!/vizhome/ExportRestrictionsTracker/FoodExportRestrictionsTracker>.

13 Bangkok Post, “Vietnam Halts New Rice Export Contracts as it Reviews Stocks”, 25 March 2020 <https://www.bangkokpost.com/business/1886355/vietnam-halts-new-rice-export-contracts-as-it-reviews-stocks>.

14 A Pandey, “Will Coronavirus Spark a Wave of Food Nationalism?”, *Deutsche Welle*, 30 March 2020 <https://www.dw.com/en/will-coronavirus-spark-a-wave-of-food-nationalism/a-52952081>.

15 DD Headey and S Fan, *Reflections on the Global Food Crisis: How Did It Happen? How Has It Hurt? And How Can We Prevent the Next One?* (Washington, DC, International Food Policy Research Institute 2011). Back in 2011, the authors noted that similar shocks are likely to reoccur in the future.

16 WTO, FAO and WHO, “Agency Chiefs Issue Joint Call to Keep Food Trade Flowing in Response to COVID-19”, *WTO*, 31 March 2020 <https://www.wto.org/english/news_e/news20_e/igo_26mar20_e.htm>.

17 J Glauber et al, “COVID-19: Trade Restrictions Are Worst Possible Response to Safeguard Food Security”, *International Food Policy Research Institute*, 27 March 2020 <https://www.ifpri.org/blog/covid-19-trade-restrictions-are-worst-possible-response-safeguard-food-security>.

18 L’Obs, “Coronavirus: Le Pouvoir d’Achat des Français sera Touché à Terme, Prévient l’Industrie Agroalimentaire”, 18 April 2020 <https://www.nouvelobs.com/coronavirus-de-wuhan/20200418.OBS27694/coronavirus-le-pouvoir-d-achat-des-francais-sera-touche-a-terme-previent-l-industrie-agroalimentaire.html>.

19 L Petetin, “The EU Common Agricultural Policy: Towards a More Sustainable Agriculture?” in R Ako and D Olawuyi (eds), *Food and Agricultural Law: Readings on Sustainable Agriculture and the Law in Nigeria* (Ado Ekiti, Afe Babalola University Press, 2015) p 201.

20 See the UN Sustainable Development Goals <https://sustainabledevelopment.un.org/?menu=1300>.

21 See, eg, Institute for European Environmental Policy, *Net-Zero Agriculture in 2050: How to Get There* (Brussels, Institute for European Environmental Policy 2019).

22 S Marlow, “COVID-19: Effects on the Fertilizer Industry”, *IHS Market*, 24 March 2020 <https://ihsmarkit.com/research-analysis/report-covid19-effects-on-the-fertilizer-industry.html>.

23 This led to an increase in household food waste. See, eg, E Royte, “Food Waste and Food Insecurity Rising Amid Coronavirus Panic”, *National Geographic*, 30 March 2020 <https://www.nationalgeographic.com/science/2020/03/food-waste-insecurity-rising-amid-coronavirus-panic>.

24 See, eg, The Poultry Site, “US Egg Prices Hit Record Levels as Pandemic Buying Boosts Demand”, 31 March 2020 <https://thepoultrysite.com/news/2020/03/us-egg-prices-hit-record-levels-as-pandemic-buying-boosts-demand>.

25 P Butler, “Food Banks Ask UK Supermarkets to Set aside Coronavirus Supplies”, *The Guardian*, 21 March 2020 <https://www.theguardian.com/society/2020/mar/21/food-banks-plead-with-uk-supermarkets-to-set-aside-supplies-amid-coronavirus-fallout>.

26 J Swinnen, “Will COVID-19 Cause another Food Crisis? An Early Review”, *International Food Policy Research Institute*, 10 April 2020 <https://www.ifpri.org/blog/will-covid-19-cause-another-food-crisis-early-review>.

27 FAO, “COVID-19: FAO and African Union Commit to Safeguarding Food Security amid Crisis”, 16 April 2020 <http://www.fao.org/news/story/en/item/1271446/icode>. See also Foodtank, “We Need a Global Stimulus Package to Avoid a COVID-19 Hunger Crisis”, April 2020 <https://foodtank.com/news/2020/04/we-need-a-global-stimulus-package-to-avoid-a-covid-19-hunger-crisis>.

28 A England et al, “G20 Agrees Debt Relief for Low Income Nations”, *The Financial Times*, 15 April 2020 <https://www.ft.com/content/5f296d54-d29e-4e87-ae7d-95ca6c0598d5>. See also World Bank Group and International Monetary Fund, “Joint Statement from the World Bank Group and the International Monetary Fund Regarding a Call to Action on the Debt of IDA Countries”, 25 March 2020 <https://www.worldbank.org/en/news/statement/2020/03/25/joint-statement-from-the-world-bank-group-and-the-international-monetary-fund-regarding-a-call-to-action-on-the-debt-of-ida-countries>.

29 This is the term used in the UK. For example, in France it is called “drive”.

30 BBC News, “Supermarkets Tesco, Asda, Aldi and Lidl Go on Hiring Spree”, 21 March 2020 <https://www.bbc.co.uk/news/business-51976075>.

31 National Farmers’ Union, “Coronavirus: NFU Seeks Crisis Meeting with Defra Secretary to Save UK’s Iconic Dairy Sector”, 8 April 2020 <https://www.nfuonline.com/news/coronavirus-updates-and-advice/coronavirus-news/coronavirus-nfu-seeks-crisis-meeting-with-defra-secretary-to-save-uks-iconic-dairy-sector>.

32 B Morton, “Coronavirus: Flour Mills Working ‘Round the Clock’ to Meet Demand”, *BBC News*, 9 April 2020 <https://www.bbc.co.uk/news/uk-52212760>.

33 The National Association of British and Irish Millers, “Retail Flour Shortages”, 1 April 2020 <http://www.nabim.org.uk/news/article/1918/retail-flour-shortages>.

34 T Morgan, “As Dairies Dump Milk, Frustration Mounts over Retail Buying Limits”, *AgWeb*, 4 April 2020 <https://www.agweb.com/article/dairies-dump-milk-frustration-mounts-over-retail-buying-limits>.

35
ibid.

36 T Lang, *Feeding Britain: Our Food Problems and How to Fix Them* (London, Penguin 2020).

37 The British food supply chain was already tested and disrupted in March 2018 when the heavy snowfall from the “Beast from the East” led to panic buying (especially milk and bread) and to missed milk collections due to blocked roads reducing the availability of milk. See, eg, S Morris et al, “Beast from the East Meets Storm Emma, Causing UK’s Worst Weather in Years”, *The Guardian*, 2 March 2018 <https://www.theguardian.com/uk-news/2018/mar/01/beast-from-east-storm-emma-uk-worst-weather-years>.

38 L Petetin, V Gravey and B Moore, “Setting the Bar for a Green Brexit in Food and Farming”, *Soil Association*, 2019 <https://www.soilassociation.org/green-brexit>.

39 National Assembly for Wales, “Loss of Access to European Single Market Potentially Disastrous for Welsh Food and Drink Industry, Says Assembly’s External Affairs Committee”, 10 December 2018 <https://www.assembly.wales/en/newhome/pages/newsitem.aspx?itemid=1943>.

40 T Lang, “Food Policy for the 21st Century” in M Koc et al (eds), *For Hunger-Proof Cities: Sustainable Urban Food Systems* (Ontario, International Development Research Centre Books 1999) p 216.

41 L Petetin, “Food Democracy in Food Systems” in PB Thompson and DM Kaplan (eds), *Encyclopedia of Food and Agricultural Ethics* (Berlin, 2^nd^ edn, Springer 2016) pp 1, 6.

42 S Booth and J Coveney, *Food Democracy: From Consumer to Food Citizen* (Berlin, Springer Briefs in Public Health 2014) p 39.

43 ND Hamilton, “Moving toward Food Democracy: Better Food, New Farmers, and the Myth of Feeding the World” *(2011) 16* Drake Journal of Agricultural Law 117, 118. For more on multilevel food governance, see, eg, L Petetin, “Managing Novel Food Technologies and Member States’ Interests: Shifting More Powers towards the Member States?” in M Varju (ed.), *Between Compliance and Particularism: Member State Interests and European Union Law* (Berlin, Springer 2019) p 233.

44 Petetin, supra, note 41, 6.

45 See, eg, in Wales the following schemes: the Welsh Food Box Company or Paul’s Organic Veg. For more information, see, eg, H Pitt et al, “C19 Horticulture Summit: Results from Edible Producers in Wales April 2020”, *Food Sense Wales*, April 2020 <http://www.foodsensewales.org.uk/pdf/C19Hort-Survey-&-Summit-Responses-3.pdf>.

46 See, eg, in Wales the following butchers: Martin Player and Oriel Jones.

47 Petetin, supra, note 41, 6.

48 C Hawkes, “Healthy Diets for Human Resilience in the Age of COVID-19”, *Nutrition Connect*, 6 April 2020 <https://nutritionconnect.org/news-events/healthy-diets-human-resilience-age-covid-19>.

49 For example, this has resulted in seed shortages in the Republic of Ireland. See, eg, F Fallon, “Coronavirus: Supply Chain Shortages Leave Gardeners Waiting for Seeds”, *The Irish Times*, 3 April 2020 <https://www.irishtimes.com/life-and-style/homes-and-property/gardens/coronavirus-supply-chain-shortages-leave-gardeners-waiting-for-seeds-1.4220286>.

50 Country Land and Business Association, “Government Clarifies Furloughing Rules after CLA Lobbying”, 7 April 2020 <https://www.cla.org.uk/government-clarifies-furloughing-rules-after-cla-lobbying>.

51 L O’Carroll, “British Workers Reject Fruit-Picking Jobs as Romanians Flown in”, *The Guardian*, 17 April 2020 <https://www.theguardian.com/environment/2020/apr/17/british-workers-reject-fruit-picking-jobs-as-romanians-flown-in-coronavirus **>**.

52 Please see “Pick for Britain” **<** www.pickforbritain.org.uk **>**. A similar platform will shortly be operational in France. See, eg, C Galtier, “Lancement d’une Plateforme pour Recruter de la Main d’Oeuvre dans l’Agroalimentaire”, *Le Figaro*, 2 April 2020 **<** https://www.lefigaro.fr/flash-eco/lancement-d-une-plateforme-pour-recruter-de-la-main-d-oeuvre-dans-l-agroalimentaire-ania-20200402 **>**.

53 L O’Carroll, “Romanian Fruit Pickers Flown to UK Amid Crisis in Farming Sector”, *The Guardian*, 15 April 2020 <https://www.theguardian.com/world/2020/apr/15/romanian-fruit-pickers-flown-uk-crisis-farming-sector-coronavirus **>**.

54 T Lang, “Food Security or Food Democracy?” (2005) 78 Pesticides News 12.

55 J Carlson and MJ Chappell, “Deepening Food Democracy”, *Institute for Agriculture and Trade Policy*, 2015 <www.iatp.org/files/2015_01_06_Agrodemocracy_JC_JC_f_0.pdf>, p 6.

56 SB Brush, *Genes in the Field: On-Farm Conservation of Crop Diversity* (Ottawa, International Development Research Centre 1999). See also M Dobbs, “Genetically Modified Crops, Agricultural Sustainability and National Opt-Outs: Enclosure as the Loophole?” (2017) 54 Common Market Law Review 1093.

57 Petetin et al, supra, note 38, 26. See also P Poux and P-M Aubert, *An Agroecological Europe in 2050: Multifunctional Agriculture for Healthy Eating – Findings from the Ten Years for Agroecology (TYFA) Modelling Exercise* (Paris, Institute for Sustainable Development and International Relations 2018).

58 See, eg, Nature Friendly Farming Network, “Feeding the Nation: How Nature Friendly Farmers are Responding to Covid-19” 2020 < https://www.nffn.org.uk/covid-19-feeding-the-nation>.

59 This is why some organisations have pushed for Food Bills to be proposed. See, eg, Sustain, “Take Action Now to Support the Household Food Insecurity Bill”, 22 January 2018 <https://www.sustainweb.org/news/jan18_food_insecurity_measurement_bill>.

60 European Commission, The Europe Green Deal, 2019, COM(2019) 640 final, 11.

61 Petetin, supra, note 41, 6.

62 N Hassanein, “Practising Food Democracy: A Pragmatic Politics of Transformation” (2003) 19(1) Journal of Rural Studies 77, 84.

